# Supplementary material for: Reproductive health needs of Human papillomavirus (HPV) positive women: A systematic review
Source: PLoS One. 2022 Sep 12;17(9):e0266819. doi: 10.1371/journal.pone.0266819 (PMC9467316; doi:10.1371/journal.pone.0266819)
Supplement: S1 File — (ZIP) [file pone.0266819.s002.zip › data availability/Response to comments .doc]

Dear Editor-in-Chief PLOS ONE

Thank you for your letter and oppertunity to revise our paper. We are ready for further revision if indicated

Best wishes

DR. Zahra Motaghi

Associate professor reproductive health

Our article is a systematic review. The data used in those articles. Therefore, the END NOTE file will be sent to use your service. However, any question or other file data is required you can contact us using the email address **galeshi_m@yahoo.com**
