# Supplementary material for: Reproductive health needs of Human papillomavirus (HPV) positive women: A systematic review
Source: PLoS One. 2022 Sep 12;17(9):e0266819. doi: 10.1371/journal.pone.0266819 (PMC9467316; doi:10.1371/journal.pone.0266819)
Supplement: Attachment — Submitted filename: Response to comments .doc [file pone.0266819.s003.doc]

Dear Editor-in-Chief

PLOS ONE

Thank you for your letter and oppertunity to revise our paper. The suggestions offered by the reviewers have been greatly helpful. We have included reviewer’s comments and responded to them individually. We hope the revised manuscript has been improved enough to be suit the PLOS ONE . We are ready for further revision if indicated

Best wishes

DR. Zahra Motaghi

Associate professor reproductive health

Reviewer# 1

1. Is the manuscript presented in an intelligible fashion and written in standard English?

*All grammatical errors typos, variations in fonts were cirrdcted throughout the paper.*

2. Abstract

*We edited*

3. Method

   In the materials and methods section, this study was conducted without language restrictions. Have you studied other languages such as Korean, French, German, etc.? While this criterion for entering this study was English and Persian.

*We edited*
Results:
1. Write a fig.1 in the findings instead of a flowchart.

*We edited*
2. In the form of a trend, the number of articles from top to bottom does not match, for example, how did the number 36 reach 1024 again? The process of selecting articles should be mentioned in detail and with details, for example, how many articles from Pabmed? How many articles from Scopus? Etc

*We edited*
3. In Tables 2 and 3, 11 articles are qualitatively evaluated, while 13 studies are included and selected

*We explain this item*

discussion
1. The first paragraph of the discussion should state exactly the main results of this study.

*We edited*
2. The findings are repeated in the discussion, while the main findings of this study should be discussed and interpreted. The main findings of this study are about the needs of women infected with papilloma virus and these needs that have been extracted in this study should be discussed and interpreted and addressed.

*We edited*
3. The strengths and limitations of this study should be mentioned at the end of the discussion

*We edited*
